# Determination of the most appropriate fertilizing method for apple trees using multi‐criteria decision‐making (MCDM) approaches

**DOI:** 10.1002/fsn3.3831

**Published:** 2023-11-20

**Authors:** Mohsen Heidarisoltanabadi, Behzad Elhami, Abdollah Imanmehr, Ali Khadivi

**Affiliations:** ^1^ Agricultural Engineering Research Department Isfahan Agricultural and Natural Resources Research and Education Center AREEO Isfahan Iran; ^2^ Department of Agricultural Machinery and Mechanization Engineering Agricultural Sciences and Natural Resources University of Khuzestan Mollasani Iran; ^3^ Department of Horticultural Sciences Faculty of Agriculture and Natural Resources Arak University Arak Iran

**Keywords:** AHP, ANP, fertilizing operation, mechanization, Semirom region, TOPSIS

## Abstract

Appropriate tree fertilization with essential nutrients is considered as one of the major factors in enhancing the quality and quantity of horticultural crops. The most efficient way to fertilize trees is to dig holes around the trunks and fill them with appropriate chemical and organic fertilizer. Doing this operation with mechanized methods reduces costs and increases productivity compared to traditional methods. In the present study, multi‐criteria decision‐making (MCDM) methods, including deterministic analytical hierarchy process (AHP) and fuzzy analytical hierarchy process (FAHP), technique for order of preference by similarity to ideal solution (TOPSIS), fuzzy TOPSIS (FTOPSIS), and analytic network process (ANP), were used to score and select the appropriate fertilizing method for apple trees based on the growers and expert's perspectives. The criteria, including fertilizing operation cost, crop yield, the percentage of tree damages, ease of entering and moving fertilizing equipment in tree rows, field capacity (with or without machinery), comfort and safety of fertilizing operations, after‐sales service, access to the required machinery and implements, crop selling price, and crop quality, were used in the above‐mentioned methods. The fertilization methods (Hole digging) considered in the present study were traditional fertilization (Shovel), orchard Trencher, motor hole digger, fixed centerline tractor‐mounted hole digger, and off‐set tractor‐mounted hole digger. Based on the results, the priority of mechanized fertilizing methods was determined as tractor‐mounted hole diggers (AHP weight of 0.286, FAHP weight of 0.285, TOPSIS relative proximity of 0.65, and FTOPSIS relative proximity of 0.64), fixed centerline tractor‐mounted hole diggers (AHP weight of 0.219, FAHP weight of 0.158, TOPSIS relative proximity of 0.56, and FTOPSIS relative proximity of 0.62), motor hole diggers (AHP weight of 0.171, FAHP weight of 0.079, TOPSIS relative proximity of 0.46, and FTOPSIS relative proximity of 0.31), and orchard trenchers (AHP weight of 0.12, FAHP weight of 0.057, TOPSIS relative proximity of 0.19, and FTOPSIS relative proximity of 0.20), respectively. Based on the ANP method, off‐set and fixed centerline tractor‐mounted hole diggers had the highest priority (weights of 0.43 and 0.27), followed by trencher (weight of 0.16), motor hole diggers (weight of 0.09), and the traditional method (weight of 0.04). Results showed that applying orchard tractors equipped with mounted diggers, especially off‐set types, can play an important role in enhancing the quantity and quality of apples produced, as well as reducing the costs of fertilizing operations.

## Graphical abstract

Based on the ANP method, off‐set and fixed centerline tractor‐mounted hole diggers had the highest priority (weights of 0.43 and 0.27), followed by trencher (weight of 0.16), motor hole diggers (weight of 0.09), and traditional method (weight of 0.04). Results showed that applying orchard tractors equipped with mounted diggers, especially off‐set types, can play an important role in enhancing the quantity and quality of apples produced, as well as reducing the costs of fertilizing operations.

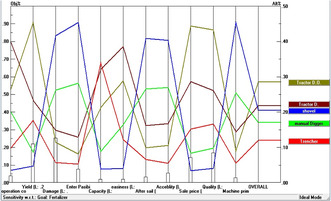

## 1 INTRODUCTION

Horticultural productions, as an important part of agricultural activities, play an important and effective role in food security and community health. However, the sustainable production of horticultural crops is threatened by environmental and managerial limitations. Tree fertilizing is one of the most important horticultural operations to improve the quality and quantity of horticultural production. Currently, the best way to fertilize trees is to dig holes around the trunks. These holes usually form on concentric circles around the tree, and their distance from the trunk of the tree depends on its species, root type, root growth pattern, and soil type. Their depth selection is based on the least damage to the roots and the maximum effectiveness of the fertilizer. The recommended amount of fertilizer is evenly distributed among the holes and, depending on the diameter of the hole, can be mixed with a variety of additives such as peat moss fertilizer, calcareous clay, perlite, gravel, sand, or superabsorbent (Gilman, 2004; Harris, 1992; Smalley & Wood, 1995). Lack of gardener knowledge about the use of suitable machines for digging holes in orchards has caused them to spread chemical and organic fertilizers on the orchard surface and mix it with the soil surface layer. This leads to the rapid decomposition of organic fertilizers and reduces the efficiency of fertilizer use. The most common form of hole diggers are those equipped with helical drills, which include different types of hand motor diggers, mini louder diggers, and tractor‐mounted diggers, in which the force is transmitted to the helical drill by a mechanical or hydraulic transmission system. By replacing the drills with different lengths, holes with a diameter of 10–20 cm and a depth of 30–100 cm can be drilled with this device (Jurgensen et al., 1997). The trencher is one of the machines used for digging continuous canals. These canals can be used to install pipes and installations, plant seedlings, or fertilize trees. The most important problem with using a hand motor digger is the possible dangers due to the drill getting stuck in the hole and applying the engine reaction force to the operator, which sometimes causes casualties (Miller et al., 2004). Other disadvantages of these mites include inefficiency in dry and heavily textured soils, a lack of ergonomic needs for users (in harsh working conditions), and scattered excavated soil around (Jurgensen et al., 1997). In a study, a self‐propelled machine similar to the mini louder digger was developed and introduced for use in dense gardens. Due to its small size and high maneuverability, this prototype can be a suitable option for digging holes and fertilizing trees in limited spaces (Taki & Asad, 2018). Accordingly, if horticultural operations (such as fertilization) could be classified into mechanized, semi‐mechanized, and traditional systems, this question might be raised about how the most suitable system can be selected and promoted for apple tree fertilizing, considering the real and scientific criteria. Given the hypothesis considered in the present study, after collecting comprehensive information and data on fertilizing methods applied in apple orchards in the Semirom region, Iran, the most appropriate and optimal mechanized fertilizing method in orchards can be determined using the analytical hierarchy process (AHP), technique for order of preference by similarity to ideal solution (TOPSIS), and analytic network process (ANP), which are called multi‐attribute decision‐making (MADM) methods. These methods have been developed to facilitate the decision‐making process (Zhang & You, 2014). The AHP method is based on three principles, including the analysis of model structure, comparative judgments, and prioritization of alternatives. Based on the model structure principle, a complicated problem is successively divided into some subcategories while considering the intended criteria, and thus a hierarchical tree structure will be created (Lin & Yang, 1996). In this method, the criteria that are more important are placed at the higher points of the hierarchical tree. Based on the second principle, pairwise comparisons are performed for all criteria, and based on that, each criterion is assigned a weight. At the final step, criteria are prioritized based on their assigned weights. This method makes the decision‐making process as planned and regulated through the formulation of the selection process so that errors will be avoided during decision‐making (Vaidya & Kumar, 2006).

The TOPSIS technique is one of the MCDM methods that is used to rank and compare different criteria, select the best one, determine their differences, and finally classify them. One of the advantages of this method is that the criteria or indicators used for comparison can have different units of measurement and also have a negative and positive nature (Zhao et al., 2010). In other words, in this method, negative and positive indicators can be used in an integrated manner. The ideal solution is the one that has the most benefit and the least cost, while the solution with the highest costs and the lowest benefits is considered as non‐ideal solution.

About the criteria related to the selection of the best tree fertilizing method, some of them are correlated to each other. In this case, the problem can be solved using the ANP method. To depict the related network, at first, the problem should be depicted in the form of a network structure, in which the goal, the main criteria, and alternatives are included for the next calculations (Eshtehardian et al., 2013). In this study, the goal is to select the best fertilizing method for apple trees.

Ahadi and Ghazanfar Rad (2011) selected the best rolling stock provider by the ANP method. Kahraman et al. (2003) used a fuzzy AHP to select the best provider in the one of Turkish factories, and concluded that decision makers could determine the priority and preference for selecting a provider using fuzzy logic variables. In this study, triangle fuzzy numbers were used, and the development method was used for analyzing paired comparisons. Kilincci and Onal (2011) used the FAHP method for selecting the best provider. The selection process was based on the customers' satisfaction. Russo and Camanho (2015) conducted a systematic review of the literature on the criteria in AHP. They tried to analyze the real cases that applied AHP to evaluate how the criteria were being defined and measured. Over 33 cases selected, they mainly used literature to build the criteria and AHP, or Fuzzy AHP, to calculate their weight, while other techniques were used to evaluate alternatives. Ayağ (2007) has proposed a hybrid approach to machine‐tool selection through AHP and simulation. Mehta et al. (2011) developed a decision support system (DSS) to select a tractor and related equipment for different soils and operating conditions. Zhou et al. (2011) proposed a new comprehensive assessment method, which combines neural networks and support vector machines (SVM) based on the particle swarm optimization (PSO) algorithm. Grisso et al. (2014) used tractor test data for selecting farm tractors. García‐Alcaraz et al. (2016) proposed a hybrid and multi‐attribute approach to assess a set of agricultural tractors based on AHP and TOPSIS methods. Bol and Mohammed (2005) developed a mathematical model for farm machinery selection. Uma Devi et al. (2012) proposed an analytic hierarchy process model for selecting the best vendor among the alternatives using the AHP method. Selecting the right vendor is a crucial decision with wide‐ranging implications for a supply chain. The proposed model can help firms in the selection process of an efficient vendor. In a study conducted by Skizari Cherati (2011), the AHP method was used to select the best weeding method in rice paddy farms among different methods including motorized weeders, manual hand weeders, and chemical control of weeds. Based on this method, there are five criteria, including agronomic, energy use management, economic, social, and ergonomic criteria. Based on the obtained results, despite environmental hazards, chemical control methods had the highest priority to use. Asadpoor et al. (2016) compared two AHP and FAHP techniques for prioritizing of crop production. Results showed that rice has a higher priority compared to wheat and rapeseed, but the FAHP method showed more precision and reliability for the final prioritization of crops compared to the AHP method. AHP and TOPSIS methods were also used to select the appropriate tractor by García‐Alcaraz et al. (2016). To this end, 18 sales specialists and farmers were selected; their priorities were identified; some criteria, including the initial price, annual costs, fuel consumption, operator safety, and after‐sales service, were selected. Ali et al. (2020) applied the multi‐criteria decision‐making technique to assess the block‐level risk of cyclones in the Sundarban region of India. The weight‐based analysis reveals that nearness to the coastline and distance from the cyclone tract have the greatest vulnerability exposure; wind speed has the highest hazard score; and nearness to cyclone shelter and cyclone awareness.

Given the appropriate weather conditions for the cultivation of cool‐season crops, Iran has a privileged position for producing apples. The area under apple cultivation and total apple production are about 248,000 ha and 4 million tonnes in Iran, respectively (Asadpoor et al., 2016). The Semirom region is the major apple producer in Iran, with a total production of 8%. Using traditional methods for fertilizing trees in this region is time‐consuming, costly, and inefficient. Selecting a suitable machinery method for tree fertilizing based on multi‐attribute decision‐making (MADM) can increase efficiency and crop yield and reduce production costs. So the present study aimed to determine the most appropriate fertilizing method for apple trees among the traditional (shovel) and mechanized methods through the MCDM process, referring to experts, operators, and growers.

## 2 MATERIALS AND METHODS

### 2.1 Study area

**FIGURE 1:**
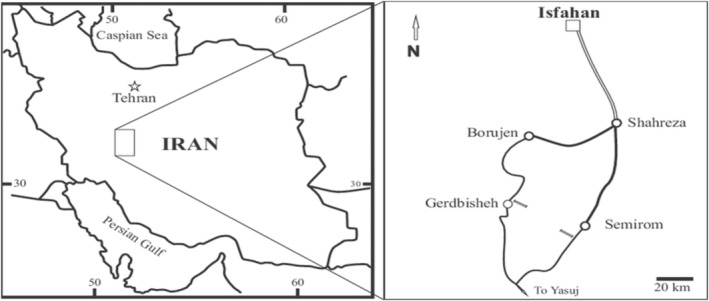
Geographical location of Semirom county in Iran ((Adapted from Naderi et al. (2019)).

The Semirom region (Figure 1) is located in the southernmost part of Isfahan province, Iran. This region, with the geographical coordinates of 51°17′ to 51°3′ eastern longitude and 30°42′ to 30°51′ northern latitude, constitutes about 10% of the total area of Isfahan province and is the major apple producer in Iran.

### 2.2 Data collection

The statistical population included apple growers, horticultural experts, and specialists. Given the large size of the target population, the random sampling method was applied. To determine the sample size, the Cochran equation (Equation 1) was applied (Kaab et al., 2019):

(1) $$n=\frac{Nz^{2}\mathrm{pq}}{Nd^{2}+z^{2}\;\mathrm{pq}\;}$$

where *N* is the statistical population (apple growers); *Z* shows the normalized value of the standard‐unit variable, which is 1.96 at a confidence level of 95%; *p* denotes the success possibility of a feature, so if there is no amount of *p*, it will be considered as 0.5; *q* shows the possibility of the failure of a feature in the population (*q* = *p*−1); and *d* is the acceptable error.

Accordingly, 45 questionnaires, including pairwise comparisons of criteria, alternatives in each criterion (based on the AHP, FAHP, and ANP methods), and scores of each criterion (based on the TOPSIS method), were randomly distributed among the apple growers and experts. Logical validity was used to determine the validity of the questionnaire, while its reliability was determined by Cronbach's alpha test (Equation 2; Gujarati, 2004).

(2) $$\alpha =\left(\frac{k}{k-1}\right)\left(1-\frac{\sum_{i=1}^{k}S_{i}^{2}}{S^{2}}\right)$$

where *k* is the number of items, *S* ^2^ is the variance of total scores related to each respondent, and $S_{i}^{2}$ is the variance of scores related to item *i*. In the present study, Cronbach's alpha was determined using SPSS V.20 software.

### 2.3 Deterministic and fuzzy analytical hierarchy process (AHP‐ FAHP)

The AHP is one of the most comprehensive systems designed for decision‐making with multiple criteria because, through this technique, the problem can be hierarchically transformed into equations and can be considered using different quantitative and qualitative criteria. This process incorporates different options in decision‐making and allows sensitivity analysis on criteria and sub‐criteria. Also, this process is based on pairwise comparison, with the possibility of facilitating judgments and calculations, and shows the degree of consistency and incompatibility of the decision. When there is not enough quantitative information and decision‐makers could not express their preferences based on quantitative and explicit judgments (uncertainty), fuzzy methods such as FAHP and FTOPSIS can provide an initial ranking of available options.

**FIGURE 2:**
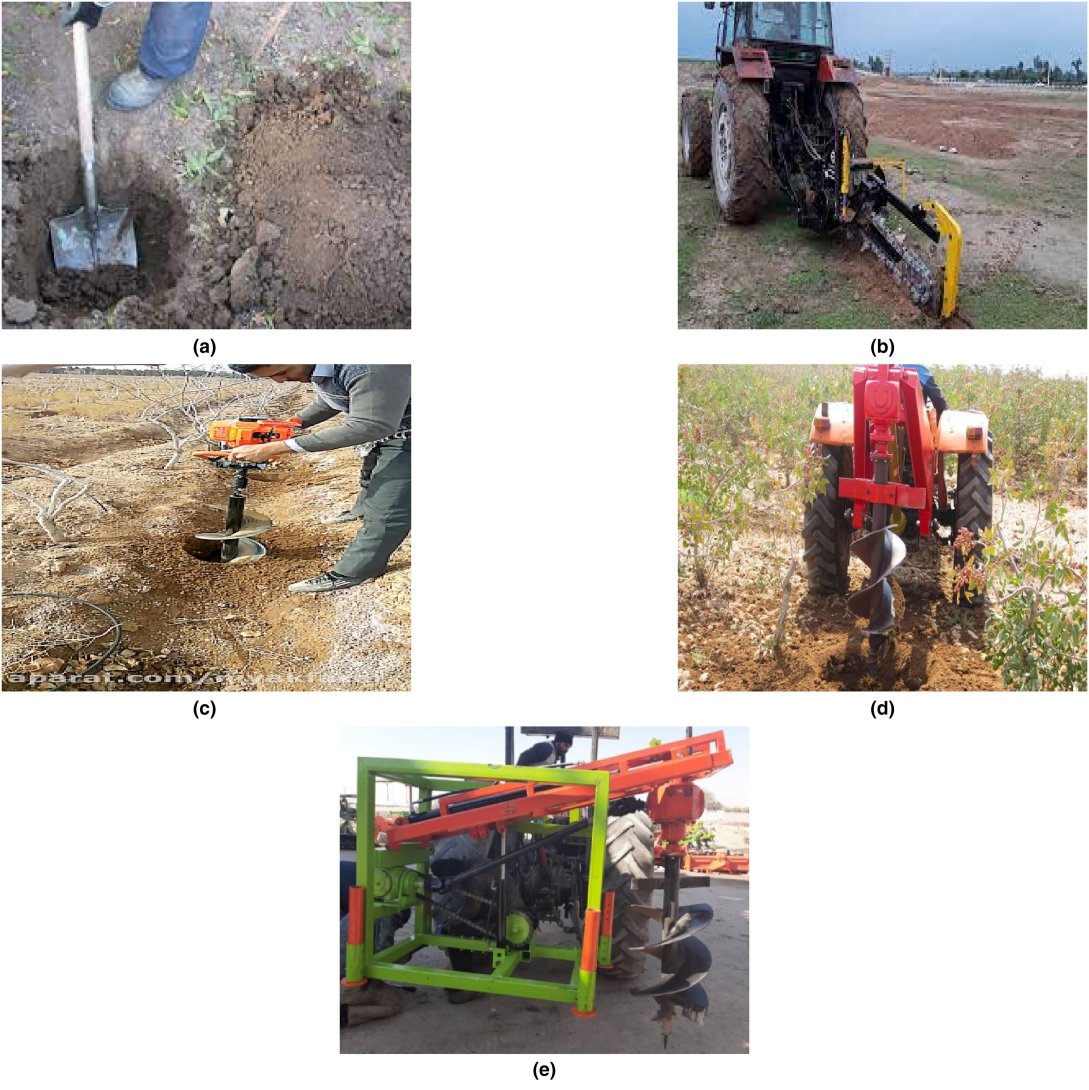
The methods of hole digging for tree fertilizing: traditional fertilizing (a), orchard trencher (b), motor hole digger (c), fixed centerline tractor‐mounted hole digger (d), and off‐set tractor‐mounted hole digger (e).

To determine the most appropriate fertilizing method for apple trees based on the AHP method, goals, criteria, and alternatives were identified. In this study, goal was identified as selecting the best fertilizing method. Also, the criteria were fertilizing operation cost, crop yield, the percentage of tree damages, ease of entering and moving fertilizing equipment in tree rows, field capacity (with or without machinery), comfort and safety of fertilizing operations, after‐sales service, access to the required machinery and implements, crop selling price, crop quality, and the original cost (initial purchase price) of fertilizing equipment. Alternatives were related to the different fertilizing methods that are applied in apple orchards in the Semirom region and were traditional fertilizing, orchard trencher, motor hole digger, fixed centerline tractor‐mounted hole digger, and off‐set tractor‐mounted hole digger (Figure 2). In the next step, pairwise comparisons were performed among the criteria, and alternatives in each criterion and then the relative weights of fertilizer methods to the criteria and criteria to each other were integrated using software or direct calculations. The result of this operation is the final weight of each fertilization method based on all criteria (Table 1). In FAHP, pairwise comparisons were performed between criteria and alternatives (in each criterion) using the preferences table and fuzzy judgments based on predefined goals, criteria, and alternatives (Table 2).

**TABLE 1:** Scale of pairwise comparison (Haralambopoulos & Polatidis, 2003).

Preferences (oral judgment)	Numerical amount
Extreme	9
Very Strong	7
Strong	5
Moderate	3
Equal	1
Intervals between strong preferences	2, 4, 6, 8

**TABLE 2:** Fuzzy equivalent of pairwise comparison (Ishizaka, 2014).

Preferences (oral judgment)	Fuzzy equivalent	Inverse fuzzy equivalent
Equal	(1, 1, 1)	(1, 1, 1)
Moderate	(1, 2, 3)	(0.33, 0.5, 1)
A little preferable	(2, 3, 4)	(0.25, 0.33, 0.5)
Moderate	(3, 4, 5)	(0.2, 0.25, 0.33)
Very preferable	(4, 5, 6)	(0.16, 0.2, 0.25)
Moderate	(5, 6, 7)	(0.14, 0.16, 0.2)
Very much preferred	(6, 7, 8)	(0.12, 0.14, 0.16)
Moderate	(7, 8, 9)	(0.11, 0.12, 0.14)
Absolutely preferred	(8, 9, 10)	(0.11, 0.11, 0.11)

**FIGURE 3:**
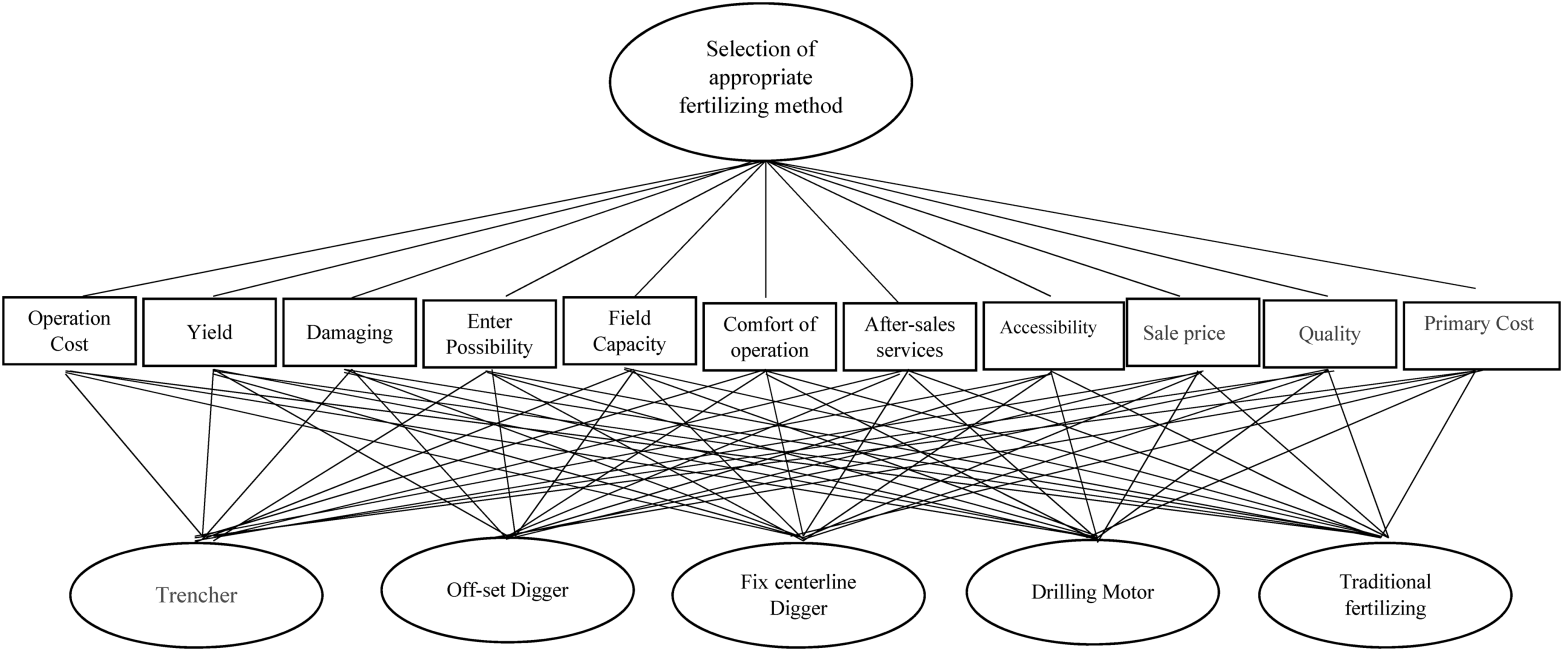
Structural arrangement of the analytical hierarchy process for the selection of an appropriate fertilization method.

Figure 3 shows the structural arrangement of AHP (deterministic and fuzzy). After a pairwise comparison of criteria and alternatives, the final weight (absolute or integrated) was calculated. All analyses related to the deterministic AHP were performed using Expert Choice software, while Microsoft excel was used for the FAHP. The inconsistency rate indicates discrepancies and inconsistencies in the pairwise comparison matrices. Based on the literature, this rate should be lower than 0.1; otherwise, the weights must be corrected to solve this problem (Saaty, 2008). The method developed by Gogus and Boucher (1998) was used to calculate the inconsistency rate of fuzzy pairwise comparison matrices.

### 2.4 TOPSIS method (deterministic and fuzzy)

The TOPSIS method was developed by Hwang et al. (1993). This method is based on the selection of the alternative that has the shortest distance from the positive ideal solution and the longest distance from the negative ideal solution. Based on this method, a problem can be solved through the following steps (Hwang et al., 1993):

1. Creating a decision matrix and converting it to a scale‐less matrix (normalized): the decision matrix included all predefined criteria and alternatives.
(3) $$n_{\mathrm{ij}}=\frac{r_{\mathrm{ij}}}{\sqrt{\sum_{i=1}^{m}r_{\mathrm{ij}}^{2}}}$$

where *r*_*ij*_ is the value of each criterion in proportion to each alternative, and *n*_*ij*_ is the value of each non‐weighted criterion.

2 Creating the weight vector of criteria (*W*), which was the same as the weights calculated in the deterministic AHP method.
3 Calculating the weighted normalized matrix: in this step, the weighted normalized matrix (*V*) was calculated using the weight vector of criteria (*W*) and Equation (4):
(4) $$V=N_{D}\times W_{m,n}$$
  where *W*_*m*,*n*_ is the weight vector of criteria and *N*_*D*_ is the value of each normalized criterion.
4 Calculating the positive and negative ideal solutions:

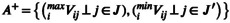


*i*= 1,2,3,…, *m*

(5) $$A_{\underset{i}{I}}^{+}=\left\{V_{1}^{+},V_{2}^{+},\ldots V_{j}^{+},\ldots ,V_{n}^{+}\right\}$$

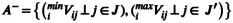




*j*= 1,2,3,…, *m*

(6) $$A_{\underset{i}{I}}^{‐}=\left\{V_{1}^{‐},V_{2}^{‐},\ldots V_{j}^{‐},\ldots ,V_{n}^{‐}\right\}$$

5 Calculating the distance:
  - Distance of alternative *i* from positive ideal solution:

(7) $$d_{i+}=\sqrt{\sum_{j=1}^{n}{\left(V_{\mathrm{ij}}-V_{j}^{+}\right)}^{2}},i=1,2,\ldots m$$

b Distance of alternative *i* from negative ideal solution:

(8) $$d_{i-}=\sqrt{\sum_{j=1}^{n}{\left(V_{\mathrm{ij}}-V_{j}^{-}\right)}^{2}},i=1,2,\ldots m$$

6 Similarity index or relative similarity to the ideal solution (Equation 9):

(9) $${\mathrm{Cl}}_{+}=\frac{d_{i-}}{d_{i+}-d_{i-}},0<{\mathrm{cl}}_{i+}<1,i=1,2,\ldots ,m$$

More similarity of alternatives to the ideal solution results in *cli* ^+^ closer to 1. Based on the descending order of *cli* ^+^, the alternatives can be ranked based on their importance level (Zavadskas et al., 2006). The criteria and alternatives defined for the AHP method were also used for the TOPSIS technique. In fuzzy TOPSIS (FTOPSIS), all calculations were done based on fuzzification of scores and weights.

### 2.5 Analytic network process (ANP) method

During the process of exploring criteria and alternatives to select the most suitable fertilizing method for trees, some criteria and alternatives are correlated to each other. Considering this issue, the ANP method was used. To draw the desired network, at first, the problem was drawn in the form of network structure, which includes the goal, main criteria, and alternatives. In this network, the goal was the selection of an appropriate method for apple tree fertilization. Then, fertilizing operation cost, yield, crop selling price (apple), and crop quality (marketing) were considered as four main criteria. Based on the growers, and expert's knowledge and perspectives, these criteria are the most effective factors in the selection of a fertilization method for apple trees, and the effectiveness of the fertilization method will be reflected in these criteria. On the other hand, these criteria are correlated with each other. For example, the selling price and the quality of fruit have a direct correlation. The alternatives considered for this method were the same as previous methods, which include traditional fertilizing (digging a hole with a shovel), fixed centerline digger, off‐set digger, orchard Trencher, and fertilizing drilling motor. To analyze the network, the selection of the most appropriate fertilization method was done based on the correlated criteria, considering the correlation (pairwise comparisons based on the fourth criterion). For example, pairwise comparisons of the apple yield, selling price, and quality criteria were done based on the fertilizing operation cost and weighting. The unweighted super‐matrix, or primary super‐matrix, was formed through the determination of criteria weights relative to each other and also the defined goal. In the next step, the weighted super‐matrix was formed, and the final weights of alternatives were determined based on the power 3 of this matrix. Finally, all methods (AHP, FAHP, TOPSIS, FTOPSIS, and ANP) were compared to each other.

## 3 RESULTS AND DISCUSSIONS

### 3.1 AHP and AHP analysis

**FIGURE 4:**
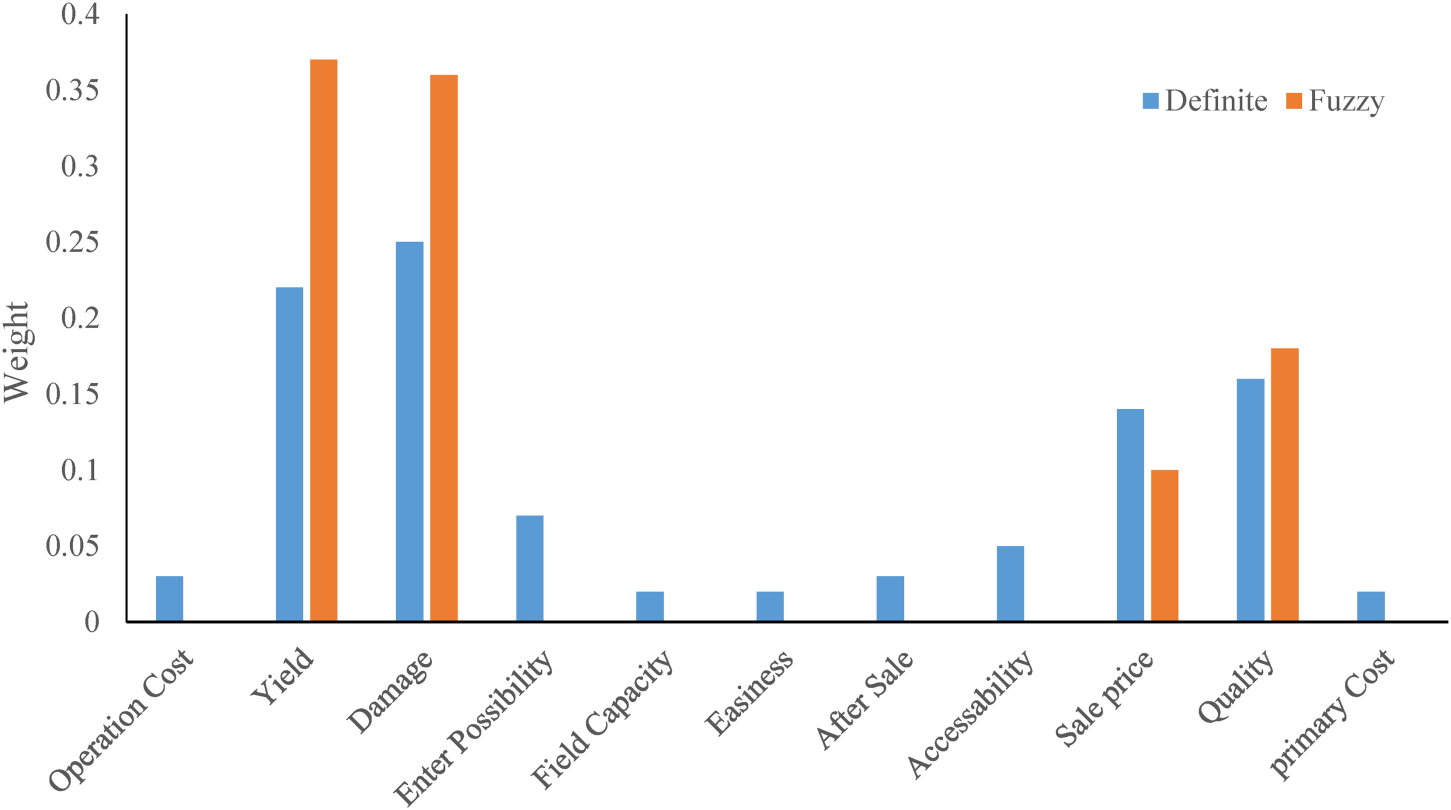
The assigned weights to the criteria considered in this study based on AHP and FAHP methods.

The criteria defined by the growers and experts for selecting the appropriate tree fertilization method in apple orchards were explored and classified based on the assigned weights to assess their preference. Figure 4 shows the assigned weights for each considered criteria. Based on the results related to AHP (shown in Figure 4), the highest and lowest weights were assigned to mechanical damage (0.25) and price and machinery capacity (0.02). Based on the FAHP results, yield and mechanical damage to trees were assigned the highest weights, with weights of 0.37 and 0.36, respectively. But 5 criteria, including fertilizing operation cost, ease of entering and moving fertilizing equipment in space between trees, comfort and safety of fertilizing operations, access to the required machinery and implements, and the original cost (initial purchase price) of fertilizing equipment, were assigned zero weight. This finding shows that, based on the expert's knowledge and perspective, mechanical damage to trees and yield of apple orchards are the most important criteria based on the AHP and FAHP methods, respectively, and so were assigned highest weights.

**FIGURE 5:**
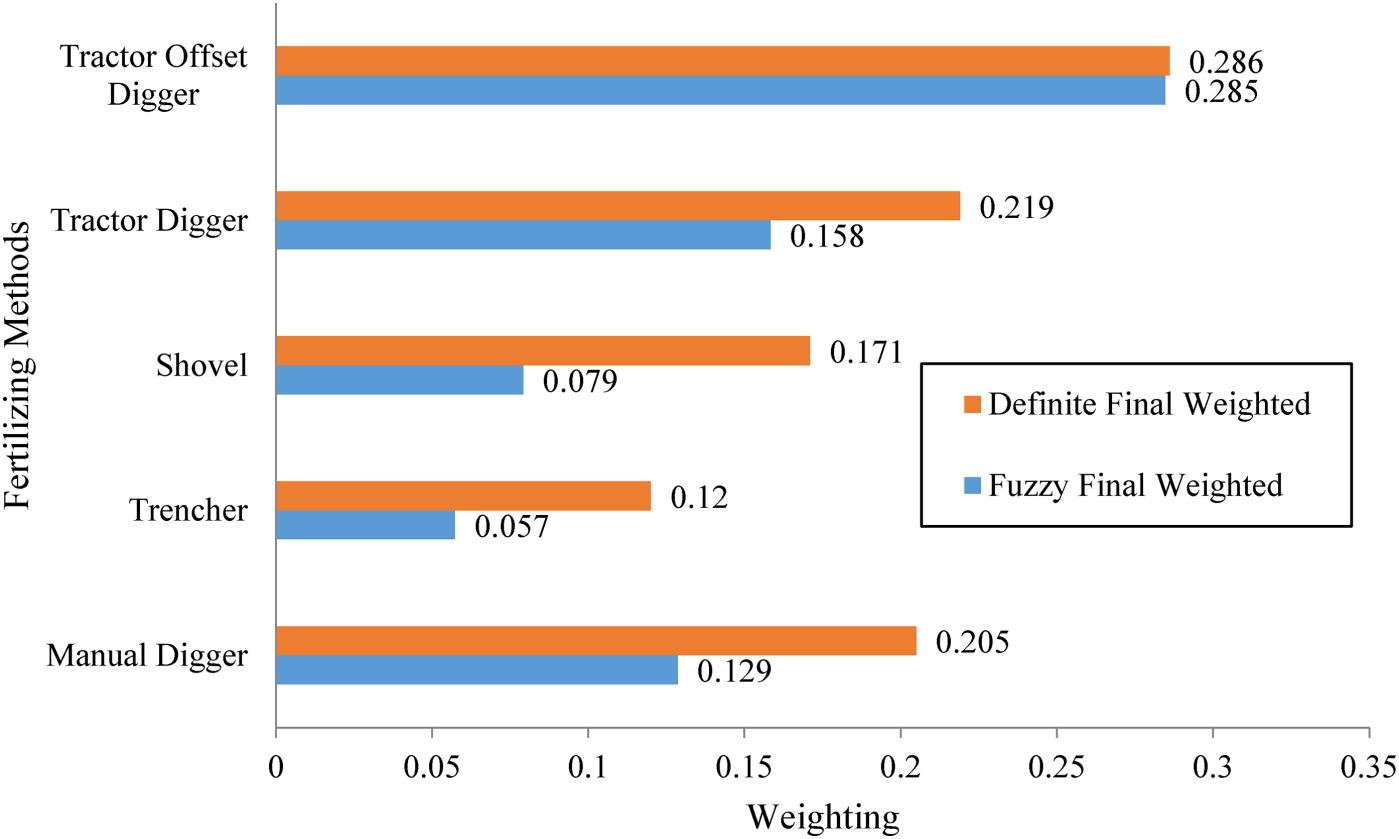
Integration of different criteria and alternatives for the selection of the most appropriate apple tree fertilization method.

**FIGURE 6:**
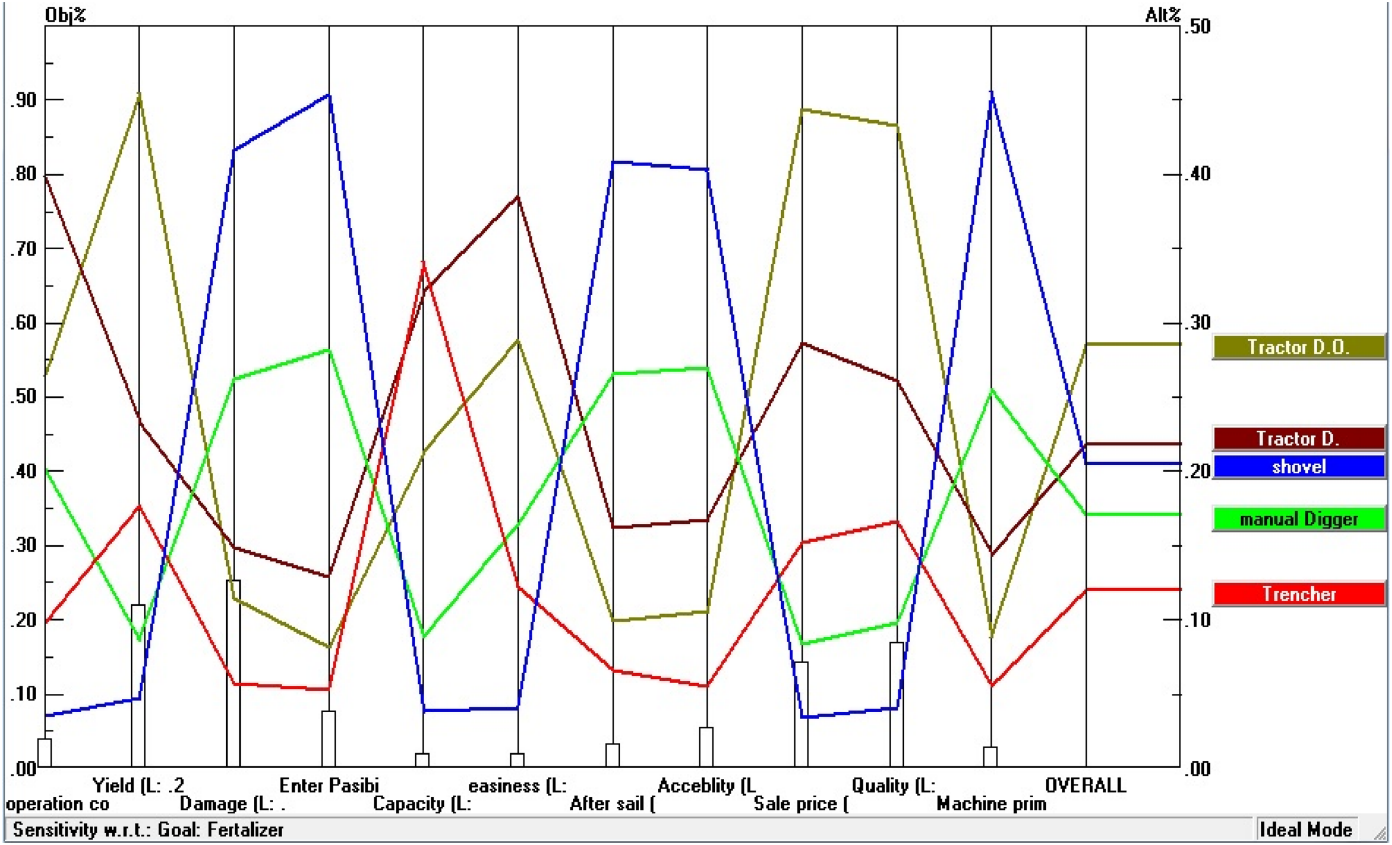
Synthesis of options and criteria according to a defined goal.

Table 3 represents the calculated weights for each of the tree fertilization methods in apple orchards according to different criteria. The traditional fertilization method included the distribution of fertilizer on the surface of the orchard or putting it in a small pit with shovels by labor, and had the lowest weight in terms of operation cost and crop selling price. This finding shows that the traditional fertilization method imposes the highest cost on the farmers, while, based on their opinions, the mechanized methods reduce the fertilization costs. Also, the traditional method has no positive impact on the crop price compared to other methods. However, the traditional fertilization method had the highest scores in some criteria, including tree damages, ease of entering space between trees, after‐sales service, access to the required machinery and implements, and the original cost (initial purchase price) of fertilizing equipment, but the preference for these criteria has not been considerable for growers. Fertilizing with a fixed centerline tractor digger was better than other methods in terms of operation cost, field capacity, comfort and safety of the fertilizing operation criteria. Based on the beneficiary's perspective, it can be attributed to the lower operational cost, higher operational speed, and ease of fertilizing operations. The offset digger was identified better in the criteria of apple orchard yield, selling price, and crop quality. Given the importance and weight of these three criteria (Figure 4), and considering the offset digger as a preferred choice, it is expected that the offset digger will be selected as the best fertilization method. The results obtained from integrating different criteria and alternatives to select the most suitable fertilization method for apple trees are shown in Figure 5. Based on these results, fertilization using an offset digger had the highest priority based on assigned weights using AHP (0.286) and FAHP (0.285). As mentioned before, this implement has a higher priority compared to other fertilizing methods in terms of orchard yield, selling price, and apple quality criteria. The fixed centerline tractor‐mounted digger had the second priority to use based on the weights of 0.219 and 0.158 using the AHP and FAHP methods, respectively. This implement had scores in some criteria, including operational costs, field capacity, and ease of fertilizing operation. The traditional fertilizing method was identified as the third priority, with weights of 0.205 (AHP) and 0.129 (FAHP). Despite high operational costs and a lower selling price of products compared to other methods, the traditional method had acceptable scores in damages to trees, ease of entering space between trees, after‐sales service, and the original price of the required implements. Fourth priority was related to the motor hole digger, with weights of 0.171 and 0.079 determined by the AHP and FAHP methods, respectively. Because of some operational problems, this implement was not popular among farmers. Orchard trencher had the lowest priority, with assigned weights of 0.12 (AHP) and 0.057 (FAHP) based on the beneficiary's perspective. The low adoption of this implement by farmers can be attributed to the difficulty of entering the implement in the space between trees, the high volume of excavation, and the higher damage rates to the extended tree roots. Figure 6 shows the sensitivity of the considered criteria to the different fertilization methods. For example, quality criterion had the highest and lowest sensitivity to offset digger and traditional method, respectively.

**TABLE 3:** Calculated weights assigned to each fertilization method for apple orchards.

Criterion/alternative	Traditional fertilizing (shovel)		Trencher		Drilling motor		Fix centerline tractor digger		Offset digger	
	Definite	Fuzzy	Definite	Fuzzy	Definite	Fuzzy	Definite	Fuzzy	Definite	Fuzzy
Operation cost	0.035	0.00	0.099	0.034	0.202	0.210	0.399	0.413	0.265	0.342
Yield	0.047	0.00	0.178	0.072	0.086	0.00	0.233	0.154	0.455	0.413
Damage	0.417	0.413	0.058	0.00	0.262	0.254	0.149	0.057	0.115	0.00
Ease of entry	0.454	0.413	0.053	0.00	0.282	0.240	0.129	0.00	0.081	0.00
Field capacity	0.038	0.00	0.341	0.344	0.088	0.00	0.320	0.413	0.212	0.380
Comfort safety	0.041	0.00	0.122	0.084	0.164	0.124	0.385	0.413	0.288	0.367
After sales services	0.408	0.413	0.066	0.00	0.265	0.309	0.162	0.160	0.098	0.00
Availability	0.402	0.413	0.055	0.00	0.270	0.340	0.167	0.201	0.105	0.042
Sale price	0.034	0.00	0.152	0.047	0.083	0.00	0.286	0.279	0.444	0.413
Quality	0.041	0.00	0.167	0.125	0.098	0.00	0.261	0.256	0.433	0.413
Cost primary	0.456	0.413	0.055	0.00	0.256	0.256	0.144	0.06	0.089	0.00

Based on the results related to the synthesis of options and criteria with consideration of the purpose of the study (Figure 5), it could be concluded that among fertilization methods in the Semirom region, the offset tractor‐mounted digger with a weight of 0.28 was identified as the most appropriate method in the study area. The inconsistency rate for all the comparisons was lower than 0.1, so criteria are totally compatible with goals and options.

### 3.2 Deterministic and fuzzy TOPSIS

Table 4 shows the distances from relative proximity to the positive and negative ideal solutions based on TOPSIS and FTOPSIS methods.

**TABLE 4:** Distances from positive and negative ideal solutions and the amounts of relative proximity based on TOPSIS and FTOPSIS methods.

Alternatives	The distance to the positive ideal		The distance to the negative ideal		Relative proximity		Rank	
	Definite	Fuzzy	Definite	Fuzzy	Definite	Fuzzy	Definite	Fuzzy
*A*_1_ (Traditional)	16.03	20.20	15.62	13.50	0.49	0.40	3	3
*A*_2_ (Trencher)	19.37	27.00	4.59	6.71	0.19	0.20	5	5
*A*_3_ (Drilling motor)	13.46	23.33	11.68	10.46	0.46	0.31	4	4
*A*_4_ (Centerline digger)	11.13	12.50	14.63	21.22	0.56	0.62	2	2
*A*_5_ (Off‐set digger)	9.00	11.93	17.32	21.78	0.65	0.64	1	1

Based on the results obtained from the TOPSIS method, fertilizing with the offset tractor‐mounted digger with the relative proximity of 0.65 (deterministic TOPSIS) and 0.64 (fuzzy TOPSIS) from the ideal solution was assigned highest priority compared to other methods. This priority can be attributed to the high weights of yield, selling price, and apple quality criteria related to this method. The second priority for tree fertilization was related to fertilizing with a fixed centerline tractor digger with the relative proximity of 0.56 and 0.62 in TOPSIS and FTOPSIS, respectively. The traditional method was the third priority, with values of 0.49 (TOPSIS), and 0.4 (FTOPSIS). As expressed in hierarchical methods, despite high operational costs and a lower selling price of products compared to other methods, the traditional method had high scores in some criteria, including damages to trees, ease of entering tree rows, after‐sales service, and the original price of required implements. Applying a motor hole digger for fertilization had low priority. The relative proximity was obtained as 0.46 and 0.31 based on the TOPSIS and FTOPSIS methods, respectively. But the lowest priority was related to fertilization with orchard trenchers, in which the relative proximity was obtained as 0.19 (TOPSIS) and 0.2 (FTOPSIS).

### 3.3 Analytic network process (ANP)

In the ANP method, four criteria, including operational costs (*C*_1_), orchard yield (*C*_2_), selling price of apples (*C*_3_), and apple quality (*C*_4_), were identified as the main criteria for selecting the most appropriate fertilization method. In Tables 5 and 6, the weighted super‐matrix and final weights related to the defined goal are shown, respectively. Based on the results obtained from this method, among these criteria, the selling price of apples (*C*_3_) was identified as the most important criterion by farmers, while orchard yield (*C*_2_), apple quality (*C*_4_), and operational costs (*C*_1_) were placed in other ranks, respectively. These findings show that the selling price of apples and yield play a major role in farmer's income. It should be noted that the selling price of a crop is affected by external factors and is out of the farmer's control. Another point is that, based on the farmer's perspective, the cost of fertilizing operations has low priority compared to other criteria.

**TABLE 5:** Weighted super‐matrix of alternatives relative to the defined goal (selection of the appropriate fertilization method).

	*A*_1_	*A*_2_	*A*_3_	*A*_4_	*A*_5_	*C*_1_	*C*_2_	*C*_3_	*C*_4_	*G*
*A*_1_	0	0	0	0	0	0.048	0.04	0.138	0.111	0
*A*_2_	0	0	0	0	0	0.095	0.2	0.172	0.148	0
*A*_3_	0	0	0	0	0	0.143	0.16	0.172	0.185	0
*A*_4_	0	0	0	0	0	0.381	0.28	0.241	0.259	0
*A*_5_	0	0	0	0	0	0.333	0.32	0.276	0.296	0
*C*_1_	0	0	0	0	0	0	0.0694	0.0556	0.0588	0.06362
*C*_2_	0	0	0	0	0	0.1976	0	0.7020	0.6905	0.5463
*C*_3_	0	0	0	0	0	0.4905	0.6325	0	0.2507	0.2411
*C*_4_	0	0	0	0	0	0.3119	0.2981	0.2424	0	0.1493
*C*_5_	0	0	0	0	0	0	0	0	0	0

**TABLE 6:** Final weight of alternatives relative to the defined goal (selection of the appropriate fertilization method).

	Alternatives					Criterion			
	*A*_5_	*A*_4_	*A*_3_	*A*_2_	*A*_1_	*C*_4_	*C*_3_	*C*_2_	*C*_1_
Weighting	0.43	0.27	0.09	0.16	0.04	0.24	0.41	0.28	0.06
Ranking	1	2	4	3	5	3	1	2	4

According to the analytic network process result, among the considered alternatives and based on the defined criteria, fertilization methods were ranked in terms of their priority of use. Based on this ranking, the fertilization methods were ordered from highest to lowest priority as follows: offset tractor‐mounted digger (*A*_5_), fixed centerline tractor‐mounted digger (*A*_4_), orchard trencher (*A*_2_), motor hole digger (*A*_3_), and traditional fertilizing (*A*_1_).

### 3.4 Comparison of fertilization methods in different decision‐making methods

Table 7 shows the ranking of different fertilization methods. Accordingly, the highest priority in AHP, FAHP, TOPSIS, and FTOPSIS methods was offset tractor‐mounted digger (*A*_5_), fixed centerline tractor‐mounted digger (*A*_4_), traditional fertilizing (*A*_1_), motor hole digger (*A*_3_), and orchard trencher (*A*_2_), respectively. According to the results, in the methods of AHP, FAHP, TOPSIS, and FTOPSIS, similar results were obtained for fertilization methods. However, the AHP method has advantages such as the ability to consider qualitative criteria in the evaluation process and having a criterion for determining the criteria weights, and disadvantages such as limiting the possibility of using a large number of criteria, the difficulty of pairing comparison process, and inconsistency between judgments. The TOPSIS and FTOPSIS methods do not have a limited number of criteria and options.

**TABLE 7:** Ranking of different fertilization methods for apple trees.

Alternatives	AHP	FAHP	TOPSIS	FTOPSIS	ANP
*A*_1_	3	3	3	3	5
*A*_2_	5	5	5	5	3
*A*_3_	4	4	4	4	4
*A*_4_	2	2	2	2	2
*A*_5_	1	1	1	1	1

In the ANP method, in which the selection criteria were limited to the 4 main criteria, including operational costs, orchard yield, selling price of apples, and apple quality, the first and second selections were obtained the same as in other methods (off‐set and fixed centerline digger), but other selections were trencher, motor hole digging, and traditional fertilization, respectively. This finding indicates that if some factors, including implement price, after‐sales service, access to technology, ease of operation, the possible damages to trees, ease of entering and moving fertilizing equipment in tree space, and field capacity of different fertilization methods, were ignored, fertilizing with trenchers and motor hole digging would have higher priority than traditional methods.

## 4 CONCLUSION

The traditional method of tree fertilization imposes high costs on farmers, and has lower efficiency compared to mechanized methods. In the present study, four multi‐attribute decision‐making (MADM) methods, including AHP, FAHP, TOPSIS, and FTOPSIS, were used to prioritize different fertilization techniques for apple trees based on the farmers, and expert's perspectives. Based on the results, 4 prioritization methods AHP, FAHP, TOPSIS, and FTOPSIS determined the priority of fertilization methods in the following order: off‐set tractor‐mounted digger, fixed centerline tractor‐mounted digger, traditional fertilizing, motor hole digging, and orchard trencher. In the ANP method, in which the criteria were limited to the 4 main criteria, including operational costs, orchard yield, selling price of apples, and apple quality, first and second selections were obtained as fertilizing with offset and fixed centerline tractor‐mounted diggers, respectively, while orchard trenching, motor hole digging, and traditional methods had lower priority. Results indicate that tractor‐mounted diggers, especially the offset type, have an important role in fertilizing apple trees according to the priorities obtained. Generally, the results of this study showed that mechanized fertilization of trees in many technical and economic aspects is better than traditional methods. Among the mechanized methods, the tractor‐mounted hole digger was selected as more appropriate than other methods due to the direct effect of digging on the quantity and quality of the produced fruit. Future studies can focus on comparing the two selected methods with modern methods.

## AUTHOR CONTRIBUTIONS

**Mohsen Heidarisoltanabadi:** Investigation (equal). **Behzad Elhami:** Investigation (equal). **Abdollah Imanmehr:** Investigation (equal). **Ali Khadivi:** Investigation (equal).

## FUNDING INFORMATION

Not applicable.

## CONFLICT OF INTEREST STATEMENT

None.

## Data Availability

The data that support the findings of this study are available from the corresponding author upon reasonable request.
